# Uncovering the associative mediation pathway from EUS features to malignancy through pathological mediators in gastrointestinal stromal tumor

**DOI:** 10.3389/fmed.2026.1803477

**Published:** 2026-07-30

**Authors:** Shaoyan Xia, Yutong Sun, Qianqian Chen, Jichao Pang, Jiafeng Wang, Wanting Li, Yaoqian Yuan, Enqiang Linghu

**Affiliations:** 1 Division of Medical Technology, Tianjin Medical University, Tianjin, China; 2 Department of Gastroenterology, Chinese People’s Liberation Army Medical School, Beijing, China; 3 Department of Gastroenterology, The First Medical Center of the People’s Liberation Army General Hospital, Beijing, China; 4 Department of Medical Imaging, The First Medical Center of the People’s Liberation Army General Hospital, Beijing, China; 5 The School of Medicine, Nankai University, Tianjin, China

**Keywords:** endoscopic ultrasound, gastrointestinal stromal tumor, graphical lasso modeling, mediation analysis, risk stratification

## Abstract

**Background:**

Endoscopic ultrasound (EUS) plays a pivotal role in preoperative risk stratification of gastrointestinal stromal tumors (GISTs); however, the biological mechanisms underlying the association between EUS imaging features and malignant risk remain incompletely understood. Most existing studies focus on predictive performance, with limited exploration of interpretable pathways linking imaging phenotypes, pathological biomarkers, and risk classification.

**Methods:**

We proposed an integrated, network-driven analytical framework to investigate the mechanistic pathways underlying GIST risk stratification. Graphical Lasso modeling was first applied to construct a sparse feature interaction network incorporating EUS and pathological variables, enabling network-based feature selection through composite influence scores derived from topological and outcome-related metrics. Subsequently, counterfactual mediation analysis was performed to systematically evaluate whether pathological biomarkers mediated the associations between key EUS features and GIST risk category. Indirect effects were assessed using stratified bootstrap with 2000 resamples, and mediation proportions were quantified to characterize the relative contribution of mediating pathways. False discovery rate (FDR) correction was applied for multiple comparisons across all exposure–mediator pathways.

**Results:**

Graphical Lasso modeling identified maximum tumor size, shape regularity, and ulceration as highly influential EUS features within the interaction network. Mediation analysis revealed that Ki-67 was the only pathological biomarker exhibiting consistent and statistically significant mediation effects across multiple EUS features (FDR < 0.05). Specifically, Ki-67 significantly mediated the effects of maximum tumor size (13.1% of total effect), shape regularity (19.73%), and ulceration (21%) on GIST risk category. In contrast, other pathological markers—including CD34, CD117, and Dog-1—did not demonstrate significant indirect effects. These findings indicate that tumor proliferative activity, rather than immunophenotypic expression alone, serves as the primary biological conduit linking EUS phenotypes to malignant risk.

**Conclusion:**

This study provides a novel, interpretable framework that integrates network-based feature discovery with counterfactual mediation analysis to uncover biologically meaningful pathways in GIST risk stratification. The results highlight Ki-67 as a central mediator bridging EUS imaging features and malignant potential, offering mechanistic insights beyond conventional predictive modeling. This approach may facilitate more transparent imaging-based risk assessment and support precision decision-making in the management of GISTs.

## 1 Introduction

Gastrointestinal stromal tumors (GISTs) are mesenchymal neoplasms characterized by striking heterogeneity in tumor behavior, ranging from indolent lesions detected incidentally to highly aggressive malignancies with substantial risks of recurrence and metastasis. Recent population-based analyses indicate that the incidence of gastrointestinal stromal tumor (GIST) continues to rise globally, while survival outcomes remain strongly influenced by tumor site, biology, and patient-related factors (1, 2). Contemporary clinical guidelines emphasize risk-adapted management based on integrated assessment of tumor size, mitotic activity, anatomical location, and mutational status, reflecting the multifactorial determinants of malignant potential (3, 4). In this context, understanding how observable tumor characteristics—rather than single variables alone—encode malignant behavior has become an important research priority, particularly for improving preoperative decision-making when definitive histopathology is not yet available.

Endoscopic ultrasonography (EUS) is the cornerstone modality for the evaluation of gastrointestinal subepithelial lesions and plays a central role in the work-up of suspected GIST. The European Society of Gastrointestinal Endoscopy (ESGE) guidelines explicitly recommend EUS for detailed characterization of lesion size, location, originating layer, echogenicity, homogeneity, borders, and growth pattern in subepithelial lesions, including GIST (5). Recent EUS-focused reviews further summarize typical GIST phenotypes such as hypoechoic texture, fourth-layer origin, irregular or lobulated margins, cystic or necrotic areas, and surface ulceration, and discuss how these features contribute to differentiating GIST from other subepithelial tumors and to estimating malignant potential (6, 7). Building on this, several groups have developed EUS image–based machine-learning or artificial intelligence (AI) models to improve diagnostic performance and risk assessment in GIST. For example, AI-assisted systems using conventional EUS images or contrast-enhanced techniques to distinguish GIST from leiomyoma and other subepithelial lesions (8, 9), or to stratify gastric GISTs into different risk categories (10, 11). Collectively, these studies highlight that EUS phenotypes carry rich information about tumor aggressiveness, but they typically treat EUS as a predictive “black box” without explicitly linking EUS patterns to downstream pathological pathways.

In parallel, pathological biomarkers remain the gold standard for biological characterization of GIST. Beyond CD117 and DOG1 expression, the proliferation marker Ki-67 has attracted growing interest as a complementary indicator of recurrence risk and survival. Recent meta-analyses and cohort studies confirm that higher Ki-67 labeling index is associated with more advanced risk categories, adverse clinicopathologic features, and poorer outcomes, suggesting its value as a prognostic and risk-stratification biomarker (12, 13). At the same time, there has been a rapid expansion of imaging–pathology research that uses CT-based radiomics or other quantitative imaging features to predict Ki-67 status and related pathological characteristics preoperatively (14–16). Multi-center and single-center studies have built radiomics nomograms to predict Ki-67 index or proliferation state in GIST, achieving moderate to high discriminative performance and demonstrating robust associations between imaging heterogeneity and underlying proliferative activity (17–21). More recently, systematic reviews and interpretable machine-learning models have further consolidated the role of quantitative imaging surrogates for Ki-67, reinforcing the concept that radiologic phenotypes can be understood as macroscopic reflections of microscopic tumor biology (22, 23).

However, most existing EUS-based and radiomics-based studies have been designed from a prediction-oriented perspective, focusing either on (i) how well imaging features distinguish GIST from non-GIST or stratify malignant risk (24–27), or (ii) how accurately imaging can approximate pathological markers such as Ki-67 (28–30). Much less attention has been paid to the associative structure linking these domains—that is, whether pathological biomarkers actually mediate the association between specific EUS features and malignant behavior in GIST. From a mechanistic standpoint, EUS-visible traits such as shape, size, surface ulceration, echotexture, and growth pattern are likely to be downstream manifestations of deeper pathological processes, including proliferative activity, cellular atypia, stromal degeneration, and microvascular alterations. Explicitly quantifying the extent to which pathological indicators mediate the relationship between EUS phenotypes and malignancy would therefore move beyond pure prediction and help transform EUS from an empirical descriptive tool into a biologically interpretable window on tumor behavior.

In this study, we aim to uncover the associative mediation pathway linking EUS imaging features, pathological biomarkers, and malignancy stratification in GISTs. Using a mediation-analysis framework, we quantify both the direct effects of key EUS features on GIST malignancy grades and the indirect effects transmitted through pathological mediators, including proliferative and immunohistochemical markers. By formally modeling the pathway *EUS features → pathology → malignancy*, our work addresses a critical knowledge gap in current GIST research: rather than asking only whether EUS can “predict” malignancy, we ask *which EUS traits genuinely reflect aggressive tumor biology via identifiable pathological mechanisms, and to what extent*. To our knowledge, this is the first study to evaluate the imaging–pathology–malignancy axis in GIST using associative mediation analysis, providing a mechanistically grounded framework that may enhance risk assessment and support more individualized management strategies. The main contributions of this paper can be summarized as follows:

1. We propose a network-driven feature discovery framework based on Graphical Lasso to characterize conditional dependency structures among EUS imaging features and pathological biomarkers in GIST.
2. We integrate counterfactual mediation analysis with network-based feature selection to establish a structured analytical pipeline for testing and quantifying whether pathological markers mediate the association between imaging phenotypes and GIST risk category, allowing direct estimation of mediation proportions at the pathway level.
3. We provide mechanistic and clinically interpretable evidence that tumor proliferative activity—indexed by Ki-67—serves as the principal biological bridge linking EUS imaging characteristics (particularly tumor size, shape regularity, and ulceration) to malignant potential, whereas diagnostic markers such as CD34, CD117, and Dog-1 do not exhibit significant mediation effects, offering actionable insights for biology-informed risk stratification.

## 2 Methods

### 2.1 Study design

**Figure 1:**
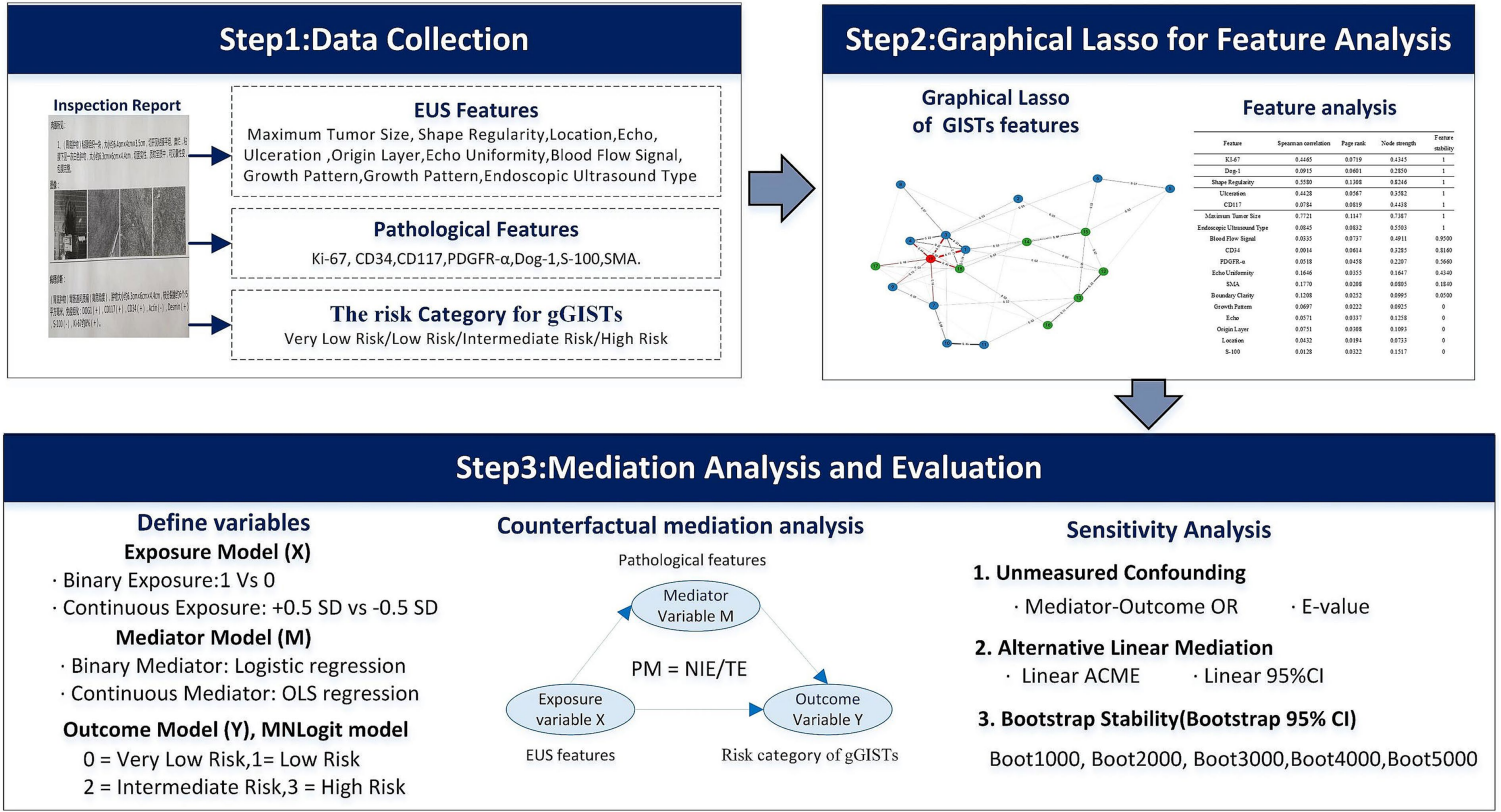
Overview of the study workflow integrating EUS feature extraction, feature selection, and mediation analysis in GISTs.

An overview of the study workflow is presented in Figure 1. This study was conducted in three major phases. In the first phase, detailed EUS findings and postoperative pathological characteristics of GISTs were systematically collected from clinical inspection reports. The EUS dataset included descriptors such as tumor size, contour regularity, echogenicity, ulceration, blood-flow signal, originating layer, and growth pattern. Corresponding pathological information—such as Ki-67 index, CD117, CD34, PDGFR-*α*, Dog-1, S-100 and SMA—was extracted from histopathology records. All variables were organized into a structured dataset along with the malignant risk categories of GISTs for subsequent analyses. The second phase involved identifying core features associated with GIST malignancy via a graphical Lasso–based feature network. After data preprocessing, the graphical Lasso algorithm was applied to estimate partial-correlation relationships among all EUS and pathological variables. Features with strong connectivity or high network influence were screened as candidates for further associative mediation analysis. In the final phase, we employed counterfactual mediation analysis to examine whether pathological biomarkers act as intermediate variables in the pathway linking EUS characteristics to GIST malignancy.

### 2.2 Study population

Our retrospective study included gastric stromal tumor patients who visited the First Medical Center of the PLA General Hospital from January 2010 to August 2023. A total of 482 gGIST patients were screened for inclusion in our retrospective study. Among them, 49 cases were excluded due to incomplete clinical or pathological data. Consequently, 433 patients with complete data for all EUS features, pathological markers, and risk category were included in the final analysis. This cohort comprised 118 patients in the very-low-risk group, 176 in the low-risk group, 84 in the intermediate-risk group, and 55 in the high-risk group. All 433 patients were used in the Graphical Lasso network analysis (feature selection stage) and the subsequent counterfactual mediation analysis, as no missing data were present in the final analytic dataset.

The inclusion criteria for patients were as follows:

1. Primary gGIST confirmed pathologically by endoscopic resection, surgery or fine-needle aspiration (FNA) biopsy;
2. Complete preoperative medical records and ultrasonographic endoscopy; and.
3. Complete clinical and pathological data.

The exclusion criteria were as follows:

1. Pathologically confirmed nongastric GIST patients;
2. A history of malignant tumors or the concurrent presence of other malignant tumors; or.
3. Incomplete clinical, imaging, or pathological information.

EUS features of the patient included the maximum cross-sectional tumor size, location, shape regularity, ulceration, originating layer, echogenicity, homogeneity of echogenicity, growth pattern, and clarity of the tumor boundary, EUS type, and blood flow signals. In addition, pathological features extracted from postoperative pathology reports included Ki-67 proliferation index, CD117, DOG-1, CD34, SMA, PDGFR-*α* and S-100. The risk classification criteria for gGISTs were based on the modified NIH criteria (1), as shown in Table 1.

**Table 1:** NIH consensus classification criteria for determining the risk of an aggressive clinical course in patients with primary GIST.

Risk category	Tumor size (cm)	Mitotic count (per 50 HPF)
Very low risk	2	5
Low risk	2–5	5
Intermediate risk	5	6–10
	5–10	5
High risk	5	5
	10	Any mitotic count
	Any size	10

### 2.3 Graphical lasso modeling for feature interaction

To explore the conditional dependency structure among EUS and pathological features and to identify the most informative variables associated with GIST malignancy, a network-based feature selection strategy was designed. All continuous variables (Maximum Tumor Size and KI-67) were first standardized using z-score normalization to ensure comparability across variables measured on different scales. A graphical lasso model (31) was applied to estimate a sparse precision matrix—that is, a partial correlation matrix in which each element quantifies the direct association between two features, while statistically controlling for the influence of all other features in the dataset. To achieve this, we used the GraphicalLassoCV function from the scikit-learn library in Python. This function implements an efficient coordinate descent algorithm (mode = “cd”) and performs 5-fold cross-validation to select the optimal regularization parameter *λ* that minimizes the log-likelihood loss. Built into the algorithm, we set the convergence tolerance to tol = 1 × 10^−4^ and the maximum number of iterations to max_iter = 100. These parameter settings ensure stable and reproducible estimates for the final precision matrix.

The sparse precision matrix estimated by Graphical Lasso was transformed into a feature interaction network. Each node represented an EUS feature, a pathological marker, or the risk category outcome. Non-zero partial correlation coefficients derived from the precision matrix were used as edge weights, representing direct conditional dependencies between pairs of features after adjustment for all remaining variables. To facilitate interpretation of feature–outcome relationships, the risk category was incorporated as an additional node, and feature–risk associations were quantified using Spearman correlation analysis. Network visualization was performed in Python using the NetworkX package, with node positions generated by the Fruchterman–Reingold force-directed layout algorithm (spring_layout). The risk category node was fixed at the center of the network, while all remaining nodes were arranged according to the force-directed optimization procedure. Node colors indicated feature categories (blue, EUS features; green, pathological markers; red, risk category), and edge width was proportional to the absolute magnitude of the corresponding association. Numerical edge labels were displayed to indicate edge weights.

To quantify the structural importance of each feature within the network, multiple network-based metrics were computed: PageRank centrality (32), which measures the global influence of a feature within the network by accounting for both the number and importance of its connected neighbors. Precision-matrix importance (33, 34), calculated as the sum of absolute values in each row of the node strength, reflecting the total conditional dependency strength of a feature. In addition to network-topological properties, the clinical relevance of each feature was evaluated by computing the absolute Spearman correlation coefficient between the feature and the ordinal malignancy risk category.

To systematically eliminate potential selection bias arising from any single, fixed weighting scheme of these three complementary metrics, a robust random-weight consensus framework was developed. Specifically, a hyperparameter sensitivity analysis was implemented by generating 500 independent weight vectors from a symmetric Dirichlet distribution, Dirichlet(1, 1, 1), which uniformly covers the entire probability simplex satisfying *w₁* + *w₂* + *w₃* = 1 (*w_i_* ≥ 0). For each random weight combination, an integrated composite influence score was calculated as the weighted combination of the normalized absolute Spearmbn an correlation, PageRank centrality, and Node strength.

Features were ranked in descending order according to the composite score within each simulation iteration. To quantify the robust hierarchy of all evaluated variables, we calculated the selection probability for each feature, defined as its empirical frequency of appearing within the top 10 highest-scoring variables across all 500 simulated Dirichlet random-weighting profiles. All features were then positioned into a generalized global stability ranking based on these consensus selection probabilities. The most highly stable features emerging from this random-weight consensus distribution were subsequently designated as the core exposure features and biological mediators for the downstream counterfactual mediation analysis.

### 2.4 Mediation analysis

To investigate whether pathological biomarkers mediate the effect of EUS features on the risk of malignant GIST (an ordinal outcome with four levels: 0 = very low, 1 = low, 2 = intermediate, 3 = high), we employed a counterfactual associative mediation framework. This approach avoids the proportional odds assumption required by ordinal logistic regression and decomposes the total effect (TE) into a natural indirect effect (NIE) that operates through the mediator and a natural direct effect (NDE) that does not, with the identity *TE* = *NIE*+*NDE.* For each exposure–mediator pair (11 EUS features × 7 pathological markers), the exposure contrast was defined as follows: for the continuous variable Maximum Tumor Size (standardized to z score) we compared a high value of +0.5 (one standard deviation increase) with a low value of −0.5 (one standard deviation decrease); for binary exposures (e.g., Ulceration, Shape Regularity, Echo) we compared 1 versus 0. All continuous variables were standardized to z scores prior to analysis, and binary mediators (CD117, PDGFR-*α*, Dog-1, SMA, S-100) were kept as 0/1.

For each bootstrap sample (see below), we first fitted a mediator model (path a ): $M=\alpha_{a}+\mathrm{aX}+\varepsilon_{a}$ , using ordinary least squares for the continuous mediator KI-67 and logistic regression for binary mediators. Then we fitted an outcome model using multinomial logistic regression:

$\log\frac{P(Y=i)}{P(Y=0)}=\alpha_{\mathrm{bj}}+c_{j}^{'}X+b_{j}M,j=1,2,3$

where *Y*=0 is the reference category. On the resampled data, we predicted the mediator values under the high and low exposure settings, denoted $M^{\text{high}}$ and $M^{\mathrm{low}}$ , and then predicted the expected outcomes for the counterfactual scenarios $Y(X=1,M^{\text{high}})$ , $Y(X=1,M^{\mathrm{low}})\;$ and $Y(X=0,M^{\mathrm{low}})$ . The effects were then computed as sample averages:

NIE=E[Y(X=1,Mhigh)−Y(X=1,Mlow)]

NDE=E[Y(X=1,Mlow)−Y(X=0,Mlow)]

TE=E[Y(X=1,Mhigh)−Y(X=0,Mlow)]

The proportion mediated was defined as PM=NIE/TE ; values outside the [0,1] interval is possible when TE is small or when NIE and NDE have opposite signs, but such cases were only reported and not interpreted for non-significant pathways.

Statistical inference was based on a stratified non-parametric bootstrap with replacement, stratifying by the outcome variable (risk categories 0–3) to preserve the original class distribution. The number of resamples was set to 2000 for the main analysis, and additional sensitivity tests used 1,000, 3,000, 4,000 and 5,000 resamples. In each bootstrap iteration, both the mediator and outcome models were refitted, and the counterfactual effects were recalculated. Point estimates were taken as the mean of the bootstrap distribution, and 95% confidence intervals were obtained as the 2.5th and 97.5th percentiles (percentile method). Bias-corrected and accelerated intervals were initially attempted but failed on several pathways due to extreme bootstrap distributions; the percentile method was robust and always computable. A two-sided *p*-value for the indirect effect was derived from its bootstrap distribution as p=min{Pr(NIE<0),Pr(NIE>0)} . Because we tested 77 exposure–mediator pathways simultaneously, we applied the Benjamini–Hochberg false discovery rate (FDR) procedure to the bootstrap *p*-values of the indirect effect; a pathway was considered statistically significant if the FDR-adjusted p-value was < 0.05.

Three sensitivity analyses were performed to evaluate the robustness of our findings. First, to quantify the potential impact of unmeasured confounding, we calculated the E-value (35) for the mediator–outcome odds ratio (path b ) of each significant pathway. Second, as a check on model specification, we repeated the entire mediation analysis treating the ordinal outcome as a continuous variable (0–3) and using ordinary least squares for the outcome model. Third, to assess the stability of the confidence intervals with respect to the number of bootstrap resamples, we re-estimated the indirect effect for the significant pathways using 1,000, 2000, 3,000, 4,000 and 5,000 resamples. All analyses were implemented in Python version 3.9 using the statsmodels library); the random seed was fixed to 2026 to ensure reproducibility.

## 3 Results

### 3.1 Characteristics of study populations

*Characteristics of EUS features*: This study enrolled a cohort of 433 GIST patients, stratified into four risk groups according to established criteria: Very-Low-Risk (*n* = 118), Low-Risk (*n* = 176), Intermediate-Risk (*n* = 84), and High-Risk (*n* = 55). The baseline EUS characteristics across these groups are summarized in Table 1. A clear trend of increasing maximum tumor size was observed with escalating risk, with median diameters of 1.27 cm, 2.65 cm, 4.00 cm, and 5.00 cm in the respective groups.

Furthermore, several high-risk EUS features demonstrated a progressive increase in prevalence across risk categories. The proportion of tumors with irregular shape rose sharply from 5.9% in the Very-Low-Risk group to 81.8% in the High-Risk group. Similarly, the presence of ulceration increased markedly from 12.7 to 74.5% across the same spectrum. Other features, such as echo heterogeneity and unclear tumor boundaries, also showed an ascending trend from low-to high-risk groups. Conversely, most tumors originated from the muscularis propria layer, and a hypoechoic echo pattern was predominant across all groups. The endoscopic ultrasound type and the distribution of tumor location did not exhibit a consistent pattern across the risk strata.

*Characteristics of pathological features*: The expression profiles of key pathological biomarkers across the four GIST risk categories are detailed in Table 2. The proliferative index Ki-67 exhibited a marked stepwise elevation with increasing risk, with median values of 3, 5, 8, and 10 in the Very-Low, Low, Intermediate, and High-Risk groups, respectively, highlighting its strong association with tumor aggressiveness. Among the diagnostic immunohistochemical markers, the expression of CD117 and CD34 was consistently high (>93%) across all groups, with CD117 positivity reaching 100% in the High-Risk group. Conversely, the expression of SMA showed an inverse relationship with risk, with positive rates declining from 47.46% in the Very-Low-Risk group to 23.64% in the High-Risk group. The positivity rates for PDGFR-*α*, DOG-1, and S-100 remained relatively stable across the risk spectrum, without a clear directional trend. These findings underscore Ki-67 as the primary pathological variable whose expression level demonstrates a direct and graded correlation with established GIST risk stratification, distinguishing it from other lineage-defining markers.

**Table 2:** Comparison of EUS features between the four patient groups.

EUS Features	Very-low-risk group (*n* = 118)	Low-risk group (*n* = 176)	Intermediate-risk group (*n* = 84)	High-risk group (*n* = 55)
Maximum tumor size (median [IQR])	1.27 [1.00, 1.83]	2.65 [2.10, 3.50]	4.00 [2.75, 5.98]	5.00 [4.00, 7.00]
Location				
Cardia (*n*, %)	1 (0.9%)	8 (4.6%)	3 (3.6%)	3 (5.5%)
Fundus (*n*, %)	62 (52.6%)	78 (44.3%)	43 (51.2%)	25 (45.4%)
Gastric-fundus junction (*n*, %)	7 (5.9%)	13 (7.4%)	8 (9.5%)	4 (7.3%)
Body (*n*, %)	36 (30.5%)	50 (28.4%)	22 (26.2%)	19 (34.5%)
Angle (*n*, %)	3 (2.5%)	12 (6.8%)	2 (2.4%)	3 (5.5%)
Antrum (*n*, %)	9 (7.6%)	15 (8.5%)	6 (7.1%)	1 (1.8%)
Shape regularity				
Yes: irregular morphology (*n*, %)	7 (5.9%)	24 (13.6%)	49 (58.3%)	45 (81.8%)
No: regular morphology (*n*, %)	111 (94.1%)	152 (86.4%)	35 (41.7%)	10 (18.2%)
Ulceration				
No (*n*, %)	103 (87.3%)	137 (77.8%)	36 (42.9%)	14 (25.5%)
Yes (*n*, %)	15 (12.7%)	39 (22.2%)	48 (57.1%)	41 (74.5%)
Origin layer				
Submucosa (*n*, %)	5 (4.2%)	3 (1.7%)	2 (2.4%)	0 (0.0%)
Muscularis propria (*n*, %)	113 (95.8%)	173 (98.3%)	82 (97.6%)	55 (100.0%)
Echo				
Low	116(98.3%)	169(96.0%)	81 (96.4%)	52(94.5%)
High	2 (1.7%)	7 (4.0%)	3 (3.6%)	3 (5.5%)
Echo uniformity				
Yes (*n*, %)	12 (10.2%)	27 (15.3%)	39 (46.4%)	23 (41.8%)
No (*n*, %)	106 (89.8%)	149 (84.7%)	45 (53.6%)	32 (58.2%)
Growth pattern				
Endophytic growth (*n*, %)	71 (60.2%)	115 (65.4%)	45 (53.6%)	27 (49.1%)
Exophytic growth (*n*, %)	31 (26.3%)	40 (22.7%)	27 (32.1%)	19 (34.5%)
Mixed growth (*n*, %)	16 (13.5%)	21 (11.9%)	12 (14.3%)	9 (16.4%)
Boundary clarity				
Yes (*n*, %)	6(5.1%)	23 (13.1%)	9 (10.7%)	11 (20.0%)
No (*n*, %)	112 (94.9%)	153 (86.9%)	75 (89.3%)	44 (80.0%)
Endoscopic ultrasound type				
Small probe (*n*, %)	95 (80.5%)	138 (78.4%)	70 (83.3%)	51 (92.7%)
Linear array (*n*, %)	8 (6.8%)	16 (9.1%)	6 (7.2%)	3 (5.5%)
Radial array (*n*, %)	15 (12.7%)	22 (12.5%)	8 (9.5%)	1 (1.8%)
Blood flow signal				
No (*n*, %)	112 (94.9%)	156 (88.6%)	75 (89.3%)	52 (94.5%)
Yes (*n*, %)	6 (5.1%)	20 (11.4%)	9 (10.7%)	3 (5.5%)

### 3.2 Feature selection of graphical lasso modeling

#### 3.2.1 Global feature interaction network revealed by graphical lasso

**Figure 2:**
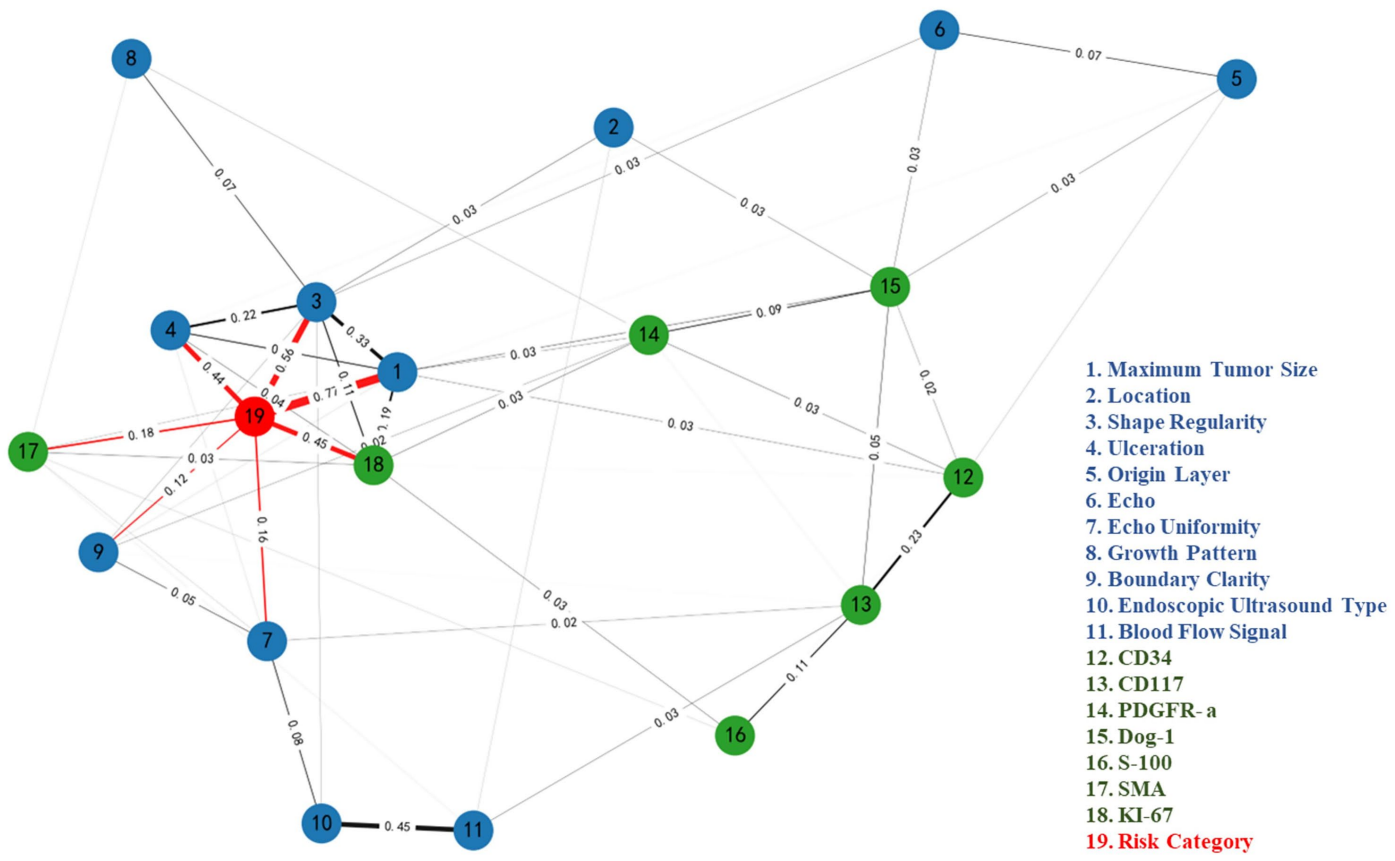
Feature interaction network via graphical lasso model.

Figure 2 presents the sparse feature interaction network derived from Graphical Lasso using all 433 patients (no missing data were present for any of the included features). Nodes represent EUS features (blue), pathological markers (green), and the risk category outcome (red). Black edges denote conditional dependencies between features estimated from the precision matrix, whereas red edges indicate feature–risk associations. Edge thickness and numerical labels correspond to the absolute magnitude of the estimated interaction strength. The resulting network exhibited a highly interconnected structure linking imaging and pathological domains. The risk category node occupied a central position and maintained direct connections with multiple EUS and pathological variables, supporting its role as the primary outcome integrating information from both feature groups. Among EUS variables, Maximum Tumor Size (Node 1) demonstrated the strongest connectivity to the risk category, showing one of the largest edge weights (0.77). Additional EUS features including Ulceration (Node 4), Shape Regularity (Node 3), and Echo Uniformity (Node 7) also displayed substantial direct associations with risk (edge weights ranging from approximately 0.10 to 0.56), suggesting that tumor morphology and internal echo characteristics are important determinants of malignant potential. Among pathological markers, Ki-67 (Node 18) showed the strongest direct association with risk (edge weight = 0.45) and was simultaneously connected to several imaging features, indicating a potential bridging role between imaging phenotype and biological aggressiveness. Other pathological SMA (Node 17) formed a tightly connected pathological subnetwork characterized by moderate conditional dependencies. Several notable feature-feature interactions were observed. Strong connections included Blood Flow Signal–Endoscopic Ultrasound Type (0.45), Ulceration–Shape Regularity (0.22), CD34–CD117 (0.23), and CD117–S-100(0.11), reflecting coordinated relationships among imaging characteristics and pathological biomarkers. Overall, the network topology suggests that tumor size, ulceration, shape regularity, and Ki-67 occupy central positions within the conditional dependency structure and may represent key candidates for subsequent mediation analysis Table 3.

**Table 3:** Comparison of pathological features between the four patient groups.

Pathological features	Very-low-risk group (*n* = 118)	Low-risk group (*n* = 176)	Intermediate-risk group (*n* = 84)	High-risk group (*n* = 55)
Ki-67 (median value)	3 [1.00, 25]	5 [0, 30]	8 [1, 40]	10 [1, 45]
SMA				
Negative (*n*, %)	62 (52.54%)	123 (69.89%)	62 (73.81%)	42 (76.36%)
Positive (*n*, %)	56 (47.46%)	53 (30.11%)	22 (26.19%)	13 (23.64%)
CD117				
Negative (*n*, %)	5 (4.24%)	11 (6.25%)	1 (1.19%)	0 (0%)
Positive (*n*, %)	113 (95.76%)	165 (93.75%)	83 (98.81%)	55 (100%)
CD34				
Negative (*n*, %)	2 (1.69%)	5 (2.84%)	0 (0%)	2 (3.64%)
Positive (*n*, %)	116 (98.31%)	171 (97.16%)	84 (100%)	53 (96.36%)
PDGFR-α				
Negative (*n*, %)	6 (5.08%)	25 (14.2%)	9 (10.71%)	5 (9.09%)
Positive (*n*, %)	112 (94.92%)	151 (85.8%)	75 (89.29%)	50 (90.91%)
S-100				
Negative (*n*, %)	107 (90.68%)	162 (92.05%)	73 (86.9%)	51 (92.73%)
Positive (*n*, %)	11 (9.32%)	14 (7.95%)	11 (13.1%)	4 (7.27%)
DOG-1				
No (*n*, %)	6 (5.08%)	21 (11.93%)	9 (10.71%)	8 (14.55%)
Positive (*n*, %)	112 (94.92%)	155 (88.07%)	75 (89.29%)	47 (85.45%)

To quantitatively prioritize features for downstream associative analysis, Table 1 summarizes the global network-based feature ranking results derived from the 500 random-weight Dirichlet simulation profiles. Overall, a distinct stratification of feature stability was observed across the entire variable spectrum. High-ranking features demonstrated remarkable structural resilience, maintaining exceptionally elevated consensus selection probabilities despite the wider random permutations of topological and clinical weights. This global probability distribution indicates that the primary prioritized variables capture intrinsic, robust dependency structures within the multivariate interaction network rather than isolated statistical artifacts. Among the endoscopic ultrasound (EUS)-derived features, maximum tumor size, ulceration, and shape regularity emerged at the apex of the global hierarchy, exhibiting the highest consensus selection probabilities. This convergence reflects their dual prominence: possessing both high-density conditional dependencies (Node strength and PageRank centrality) within the Graphical Lasso feature network and robust macroscopic associations (Spearman correlation) with the ordinal malignancy risk categories. Conversely, peripheral EUS imaging metrics demonstrated a synchronized and sharp decline in selection frequencies under random weight variations, verifying their lower structural stability. In terms of pathological and immunohistochemical markers, KI-67 and DOG-1 manifested prominent ranking positions with robust selection stability. The high consensus probability of KI-67 was heavily supported by its substantial conditional interaction density with neighboring EUS features and its direct associative alignment with high-grade malignancy risk. Other essential biomarkers, such as CD117 and CD34, also demonstrated stable, moderate-to-high global consensus profiles, establishing their critical biological roles in linking microscopic tumor biology (Node strength) to macroscopically observable imaging phenotypes Table 4.

**Table 4:** Feature analysis of graphical lasso model.

Feature	Spearman correlation	Page rank	Node strength	Feature stability
KI-67	0.4465	0.0719	0.4345	1
Dog-1	0.0915	0.0601	0.2850	1
Shape regularity	0.5580	0.1308	0.8246	1
Ulceration	0.4428	0.0567	0.3582	1
CD117	0.0784	0.0819	0.4438	1
Maximum tumor size	0.7721	0.1147	0.7387	1
Endoscopic ultrasound type	0.0845	0.0832	0.5503	1
Blood flow signal	0.0335	0.0737	0.4911	0.9500
CD34	0.0014	0.0614	0.3285	0.8160
PDGFR-α	0.0518	0.0458	0.2207	0.5660
Echo uniformity	0.1646	0.0355	0.1647	0.4340
SMA	0.1770	0.0208	0.0805	0.1840
Boundary clarity	0.1208	0.0252	0.0995	0.0500
Growth pattern	0.0697	0.0222	0.0925	0
Echo	0.0571	0.0337	0.1258	0
Origin layer	0.0751	0.0308	0.1093	0
Location	0.0432	0.0194	0.0733	0
S-100	0.0128	0.0322	0.1517	0

### 3.3 Results of mediation analysis

#### 3.3.1 Significant path analysis

**Figure 3:**
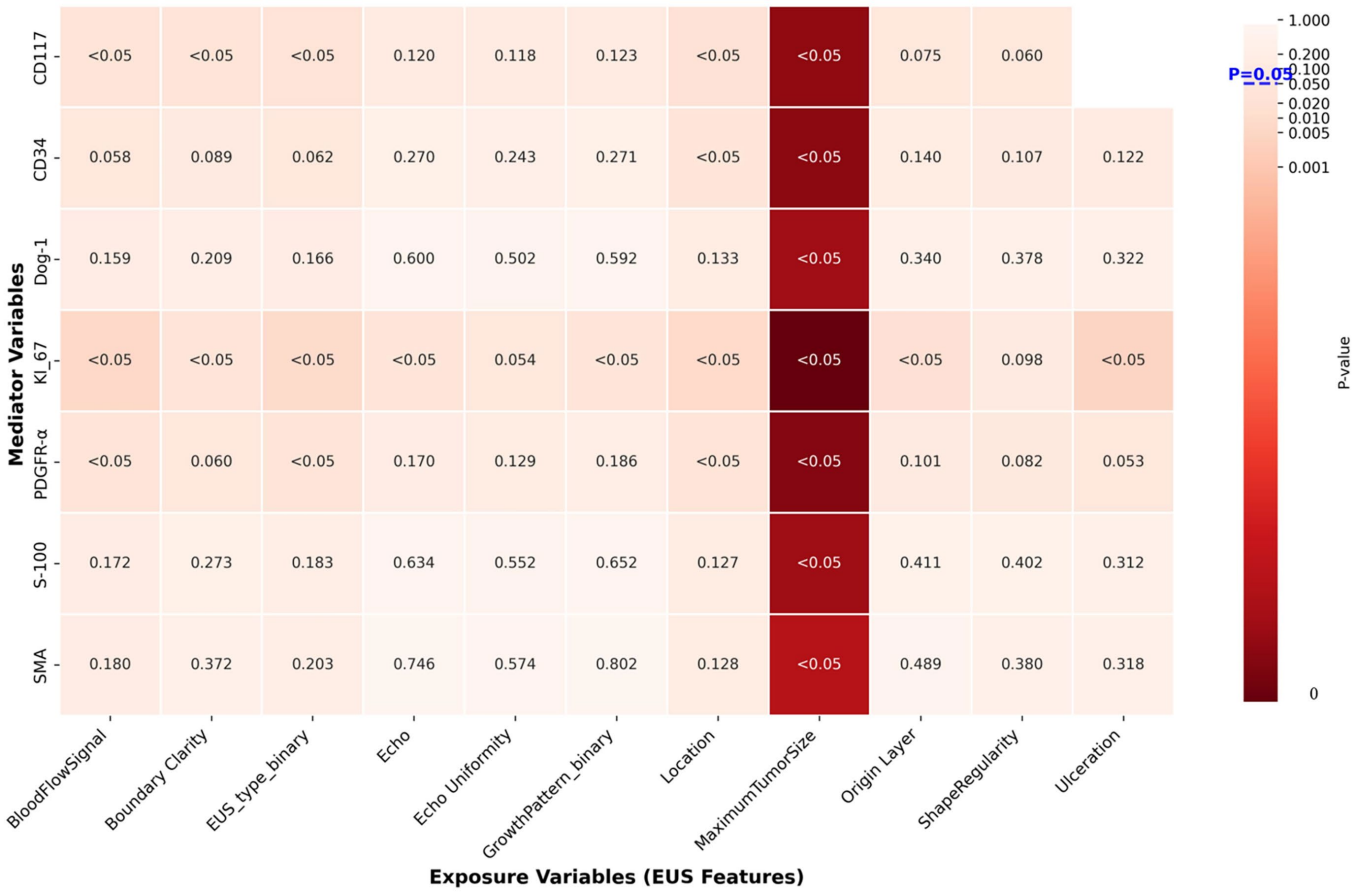
Evaluation of the proportional odds assumption across all exposure-mediator combinations via python-based likelihood ratio tests.

To rigorously evaluate the associative pathways, a counterfactual mediation analysis framework under the potential outcomes philosophy was implemented, using the full analytic cohort of 433 patients with complete data for all exposures, mediators, and the outcome variable. A critical preliminary step involved determining the most appropriate regression model for the multi-categorical ordinal outcome (Y). To formally justify our modeling choice, we conducted a comprehensive Likelihood Ratio Test (LRT) comparing a constrained ordered logistic regression (proportional odds model) against an unconstrained multinomial logistic regression for each exposure-mediator (X → M) combination. As presented in Figure 3, a substantial proportion of the tested pathways exhibited LRT *p*-values < 0.05, indicating a significant violation of the proportional odds assumption for those specific model specifications. Forcing a standard ordered logistic regression model on data where this parallel-lines constraint is violated would lead to biased and unstable parameter estimates, undermining the validity of subsequent mediation effect decomposition. Consequently, to maintain strict statistical rigor and ensure unbiased effect estimation across all pathways, we uniformly adopted Multinomial Logistic Regression (MNLogit) as the outcome model for the counterfactual mediation analysis. This model completely relaxes the proportional odds restriction, allowing the effect of predictors to vary across different levels of the ordinal outcome. The framework utilized an adaptive modeling strategy incorporating Ordinary Least Squares (OLS) linear regression or binary logistic regression for the mediators (M), depending on their distribution, and Multinomial Logistic Regression (MNLogit) to accommodate the multi-categorical ordinal outcome (Y). To account for the distribution of the outcome, a stratified bootstrapping procedure (2,000 replications) conditioned on outcome levels was utilized to compute the point estimates and 95% percentile confidence intervals (CIs) for the Total Effect (TE), Natural Direct Effect (NDE), and Natural Indirect Effect (NIE).

After applying the Benjamini-Hochberg False Discovery Rate (FDR) correction across all 77 hypothesized associative paths to control for multiple testing, the majority of the pathways did not reach statistical significance. Crucially, three distinct mediating pathways successfully passed the strict multi-test correction framework, all converging on the KI-67 proliferation index as a central biological mediator. First, a highly prominent path was confirmed from Ulceration to clinical risk category mediated by KI-67 (Ulceration → KI-67 → Risk Category). Under the primary analysis (2,000 bootstrap resamples), the Natural Indirect Effect (NIE) was estimated to be 0.2329 (95% CI: 0.0971 to 0.3940), maintaining strict significance after adjustment (*P_FDR_* < 0.001). The Total Effect (TE) and Natural Direct Effect (NDE) were 1.1092 (95% CI: 0.9108 to 1.3116) and 0.8763 (95% CI: 0.6993 to 1.0409), respectively, indicating that KI-67 expression mediated 21% of the effect of Ulceration on risk outcomes. Second, the presence of Shape Regularity exerted a significant indirect effect on risk classification via KI-67 (Shape Regularity → KI-67 → Risk Category), with an estimated NIE of 0.2706 (95% CI: 0.1513 to 0.4183; adjusted PFDR < 0.001). For this pathway, the TE was 1.3715 (95% CI: 1.2015 to 1.5300) and the NDE was 1.1009 (95% CI: 0.9340 to 1.2578), with KI-67 accounting for 19.73% of the total driving effect. Third, the Maximum Tumor Size similarly manifested a significant indirect pathway through KI-67 (Maximum Tumor Size → KI-67 → Risk Category). The NIE was identified as 0.1358 (95% CI: 0.0657 to 0.2290, adjusted PFDR < 0.001), alongside a TE of 1.0366 (95% CI: 0.9183 to 1.1658) and an NDE of 0.9009 (95% CI: 0.7911 to 1.0109). The proportion of the Maximum Tumor Size effect mediated by KI-67 was 13.1% Table 5.

**Table 5:** Significant mediation analysis of EUS features through pathological biomarkers for GIST risk stratification.

Exposure variable X	Maximum tumor size	Ulceration	Shape regularity
Mediation M	KI-67	KI-67	KI-67
TE	1.0366	1.1092	1.3715
TE CI low	0.9183	0.9108	1.2015
TE CI high	1.1658	1.3116	1.5300
NIE	0.1358	0.2329	0.2706
NIE CI low	0.0657	0.0971	0.1513
NIE CI high	0.2290	0.3940	0.4183
NDE	0.9009	0.8763	1.1009
NDE CI low	0.7911	0.6993	0.9340
NDE CI high	1.0109	1.0409	1.2578
Proportion mediation (PM)	13.1%	21%	19.73%
Indirect P	<0.001***	<0.001***	<0.001***
FDR-adjusted P	<0.001***	<0.001***	<0.001***

*** *p* < 0.001, ** *p* < 0.01, * *p* < 0.05.

#### 3.3.2 Sensitivity analysis

To assess the robustness of these Significant mediating path, sensitivity analyses concerning unmeasured confounding, model specification, and bootstrapping parameters were sequentially conducted for the three significant pathways.

Regarding sensitivity to unmeasured confounding, the estimated E-values for the mediator–outcome relationships were 68.82 for Maximum Tumor Size, 4.26 for Ulceration, and 5.07 for Shape Regularity (Table 6). All three E-values are substantially above 1, indicating that relatively strong unmeasured confounding would be needed to fully explain away the observed indirect effects. The Maximum Tumor Size pathway (*E*-value = 68.82) is exceptionally robust, while the Ulceration (4.26) and Shape Regularity (5.07) pathways also demonstrate strong robustness. Overall, the sensitivity analysis supports the robustness of the major mediation findings.

**Table 6:** Sensitivity analysis of model specification.

Path	OR (M → Y)	E-value	Linear NIE	Linear 95% CI
Maximum tumor size→KI-67	34.663	68.82	0.0837	[0.0510, 0.1258]
Ulceration→KI-67	2.412	4.26	0.1415	[0.0615, 0.2291]
Shape regularity→KI-67	2.813	5.07	0.1872	[0.1132, 0.2718]

For model specification sensitivity, an alternative framework treating the ordinal risk categories as a continuous outcome via OLS linear regression (with 2,000 bootstrap resamples) demonstrated consistent statistical significance across all three paths, yielding alternative indirect effects for maximum tumor size (Linear NIE = 0.0837, 95% CI: 0.0510 to 0.1258), ulceration (Linear NIE = 0.1415, 95% CI: 0.0615 to 0.2291), and Shape Regularity (Linear NIE = 0.1872, 95% CI: 0.1132 to 0.2718). These synchronized results confirm that the detected mediation architecture is structurally stable and not an artifact of specific multi-categorical categorical model assumptions Table 7.

**Table 7:** Bootstrap confidence interval distributions for key significant mediation paths.

Path	Maximum tumor Size→KI_67	Ulceration→KI_67	Shape regularity→KI_67
Boot1000	[0.0660, 0.2288]	[0.0938, 0.3865]	[0.1506, 0.4193]
Boot2000	[0.0683, 0.2377]	[0.0966, 0.4059]	[0.1532, 0.4180]
Boot3000	[0.0658, 0.2341]	[0.0964, 0.4036]	[0.1504, 0.4164]
Boot4000	[0.0664, 0.2353]	[0.0960, 0.3915]	[0.1493, 0.4295]
Boot5000	[0.0650, 0.2310]	[0.1009, 0.3968]	[0.1480, 0.4151]

Furthermore, evaluation across a multi-gradient bootstrap resampling scale—specifically testing 1,000, 2,000, 3,000, 4,000, and 5,000 iterations—demonstrated high numerical stability in the 95% CIs across all execution runs. The 95% CIs derived from the sequential resampling gradients (1,000, 2,000, 3,000, 4,000, and 5,000 replications, respectively) exhibited strict convergence and bounded intervals: 0.0660 to 0.2288, 0.0683 to 0.2377, 0.0658 to 0.2341, 0.0664 to 0.2353 and 0.0650 to 0.2310 for maximum tumor size; 0.0938 to 0.3865, 0.0966 to 0.4059, 0.0964 to 0.4036, 0.0960– to 0.3915, 0.1009 to 0.3968 for ulceration; and 0.1506 to 0.4193, 0.1532 to 0.4180, 0.1504 to 0.4164, 0.1493 to 0.4295, 0.1480 to 0.4151 for Shape Regularity. This absolute convergence across progressive sample sizes rules out potential computational bias driven by random resampling artifacts.

## 4 Discussion

In this study, we adopted a network-driven feature discovery strategy combined with mediation analysis to investigate the mechanistic pathways linking EUS features, pathological biomarkers, and GIST risk stratification. Using Graphical Lasso modeling, we identified a compact set of EUS and pathological variables that occupied central positions within the feature interaction network. Subsequent mediation analyses revealed that among multiple pathological biomarkers, Ki-67 uniquely and consistently mediated the associations between key EUS features (maximum tumor size, shape regularity, and ulceration) and GIST risk category, whereas other commonly used pathological markers (CD34 and Dog-1) did not demonstrate statistically significant mediation effects. These findings suggest that Ki-67 represents a critical biological bridge connecting macroscopic imaging phenotypes captured by EUS with microscopic tumor aggressiveness, providing a mechanistic explanation for why certain EUS features are strongly predictive of malignant risk.

### 4.1 Interpretation of graphical lasso–based feature selection

Graphical Lasso modeling enabled the identification of conditional dependency relationships among EUS features and pathological biomarkers after adjusting for all remaining variables. Unlike univariate or correlation-based screening, this approach emphasizes direct feature–feature interactions, allowing biologically meaningful structures to emerge.

Within the constructed network, maximum tumor size, shape regularity, ulceration, and Ki-67 exhibited high network connectivity and edge strength, indicating their central roles in the multivariate dependency structure. These features also ranked consistently high in composite importance scores integrating correlation with outcome, network topology, and node strength –based metrics. Importantly, the convergence of network centrality and downstream mediation significance supports the biological plausibility of the selected features. This network-informed selection framework thus provides a robust foundation for subsequent associative pathway analysis.

### 4.2 Ki-67 as a biological mediator linking EUS phenotypes to malignant risk

A central finding of this study is that Ki-67 acted as a significant mediator in multiple EUS–risk pathways, accounting for a substantial proportion of the total effect of imaging features on GIST risk category. Ki-67 is a well-established marker of cellular proliferation and has been incorporated into several GIST risk stratification systems, including modified NIH criteria and WHO classifications. Elevated Ki-67 expression reflects increased mitotic activity, genomic instability, and aggressive tumor biology. Recent studies have consistently shown that Ki-67 is independently associated with recurrence risk, metastatic potential, and overall survival in GIST patients (36). From an imaging–pathology perspective, the mediation role of Ki-67 is biologically intuitive. EUS features such as tumor size, shape irregularity, and ulceration represent macroscopic manifestations of underlying proliferative and invasive behavior, which are directly captured at the cellular level by Ki-67 expression.

Maximum tumor size demonstrated a strong positive association with Ki-67 expression and a significant indirect effect on risk category through Ki-67. Tumor growth in GIST is primarily driven by sustained proliferative signaling downstream of KIT or PDGFRA mutations. Larger tumors typically accumulate higher proliferative indices due to prolonged unchecked cell cycling, which is directly reflected by elevated Ki-67 labeling. Recent EUS-based radiopathological studies have reported that tumor size correlates not only with mitotic count but also with Ki-67–based proliferation indices (15, 37), reinforcing the biological continuity between imaging scale and histopathological aggressiveness. The observed mediation proportion indicates that a meaningful fraction of the size–risk association operates through tumor proliferation, rather than through size alone.

Shape regularity exhibited a significant positive association with Ki-67 and a corresponding positive indirect effect on risk category. Irregular tumor contours on EUS often reflect heterogeneous growth patterns, asymmetric expansion, necrosis, and invasive margins. These morphological features are characteristic of tumors with high proliferative heterogeneity and aggressive biological behavior. Experimental and clinical studies have shown that tumors with high Ki-67 expression frequently display architectural disorganization and invasive growth fronts, which are detectable as irregular shapes on imaging (38, 39). The mediation findings suggest that shape irregularity serves as a macroscopic surrogate for proliferative dysregulation, with Ki-67 acting as the microscopic mediator linking morphology to malignancy risk.

Ulceration showed a robust positive association with Ki-67 and a significant indirect effect on risk category. Ulceration in GIST is typically associated with rapid tumor growth, mucosal ischemia, and surface breakdown (40). These processes are frequently driven by high cellular turnover and aggressive proliferation. Clinical studies have demonstrated that ulcerated GISTs tend to exhibit higher mitotic counts, increased Ki-67 indices, and worse prognostic outcomes compared with non-ulcerated lesions (41). The mediation analysis supports the hypothesis that ulceration reflects a proliferative phenotype, with Ki-67 capturing the biological mechanism through which ulceration contributes to increased malignant risk.

In contrast to Ki-67, CD34 and Dog-1 did not demonstrate significant mediation effects despite showing weak associations with certain EUS features. This finding is biologically consistent with the functional roles of these markers. CD34 and Dog-1 are primarily diagnostic and lineage-associated markers (42), reflecting tumor origin, vascularity, or interstitial cell differentiation rather than proliferative activity (43). While these markers are essential for confirming GIST diagnosis, their expression levels are relatively stable across risk categories and do not directly capture dynamic tumor aggressiveness (44, 45). The lack of mediation observed in this study reinforces the concept that not all pathological variables are mechanistically positioned to link imaging features with malignant potential, underscoring the specificity of Ki-67 as a functional mediator.

### 4.3 Integrated interpretation of mediation patterns

Several consistent patterns emerged from the mediation analysis. First, all significant pathways exhibited partial mediation, with direct effects of EUS features remaining significant after accounting for Ki-67. This indicates that while proliferative activity explains an important component of the imaging–risk association, the majority of the effect operates through other biological pathways not captured in this study. For example, approximately 21% of the effect of ulceration on malignant risk is mediated through Ki-67 expression, meaning 79% of the effect operates through other pathways. Similarly, 13.1% of the effect of maximum tumor size and 19.73% of the effect of shape regularity are mediated through Ki-67, leaving 86.9 and 80.27%, respectively, to be explained by other mechanisms. Second, mediation proportions varied across EUS features, with ulceration (21%) demonstrating stronger mediation through Ki-67 than tumor size (13.1%) and shape regularity (19.73%), suggesting feature-specific biological relevance. The higher proportion for ulceration may reflect the biological link between mucosal disruption, local inflammation, and subsequent proliferative activation. Conversely, the direct effects of tumor size and shape regularity may reflect additional structural and geometric factors (e.g., mass effect, invasion potential) that influence risk independently of proliferation. Finally, the mediation role of Ki-67 reinforces its central position as a functional biomarker linking macroscopic imaging phenotypes to microscopic tumor aggressiveness.

Taken together, these findings provide a coherent biological framework in which specific EUS features—particularly maximum tumor size, shape regularity, and ulceration—convey malignant risk partly through their association with tumor proliferative activity, as quantified by Ki-67. This integrated network–mediation approach advances our understanding of how imaging phenotypes reflect underlying tumor biology and offers a principled basis for biologically informed risk stratification in GIST.

## 5 Conclusion

This study demonstrates that a network-driven analytical framework integrating Graphical Lasso modeling and counterfactual mediation analysis can effectively elucidate mechanistic pathways linking endoscopic ultrasound features, pathological biomarkers, and malignant risk in gastrointestinal stromal tumors. Among all evaluated pathological variables, Ki-67 emerged as the sole and consistent mediator connecting key EUS phenotypes—namely maximum tumor size, shape regularity, and ulceration—to GIST risk category. These findings indicate that the prognostic value of selected EUS features is largely conveyed through underlying tumor proliferative activity rather than through diagnostic immunohistochemical markers alone. By providing a biologically interpretable explanation for imaging-based risk stratification, this study highlights the potential of proliferation-centered mediation mechanisms to enhance clinical decision-making and supports the development of integrative imaging–pathology models for GIST risk assessment.

## Data Availability

The original contributions presented in the study are included in the article/supplementary material, further inquiries can be directed to the corresponding authors.
